# Cumulative stressor exposure and cognitive functioning in late childhood: The role of inflammation

**DOI:** 10.1111/bjdp.12557

**Published:** 2025-03-23

**Authors:** Izabella Polgar‐Wiseman, Marta Francesconi, Eirini Flouri

**Affiliations:** ^1^ Department of Psychology and Human Development, Institute of Education University College London London UK

**Keywords:** adverse life events, ALSPAC, executive function, inflammation, social communication

## Abstract

This study examined whether the experience of stressors since infancy is related to executive function and social communication in late childhood via inflammation, using data from 4457 participants of the Avon Longitudinal Study of Parents and Children (ALSPAC). It explored whether the effect of stressful life events (from 6 months to 8.5 years) on working memory, response inhibition, selective attention, attentional control, communication problems and social cognition (at ages 10–11 years) was mediated by inflammation (interleukin 6 and C‐reactive protein) at age 9 years. While the study did not find evidence for mediation, it showed that, in the general child population, inflammation was related to executive function impairments, and stressful life events were related to social communication difficulties. These associations were small but robust to confounder adjustment. If causal, they suggest that reducing inflammation could improve executive functioning, the prerequisite to any purposeful and goal‐directed action.

**Key points**

- We explored whether, in the general population, lifetime stressor exposure is related to poor cognitive functioning in children (aged 10‐11) via inflammation (IL‐6 and CRP at age 9).
- Cognitive functioning included working memory, selective attention, attentional control, response inhibition, communication problems and social cognition
- Inflammation did not mediate the link between lifetime stressor exposure and cognitive functioning
- Nonetheless, inflammation was related to executive function impairments
- Also, lifetime stressor exposure was related to social communication difficulties.

## INTRODUCTION

Although the impact of stressful life events (SLE) on child health is well established (Huang et al., 2015; Ochi & Dwivedi, 2023; Widom et al., 2012), the evidence for their role on child cognition, especially in the general population, is rather mixed (Bos, 2009; Francesconi et al., 2023; Holland et al., 2020; Nweze et al., 2023). While some studies have linked stressors experienced in childhood to impairments in cognitive functioning—showing links with both childhood IQ (Young‐Southward et al., 2020) and executive functioning and working memory in adulthood (McManus et al., 2022)—others have not found evidence of a significant relationship (Bos, 2009; Francesconi et al., 2023; Holland et al., 2020; Nweze et al., 2023). One reason for this mixed picture may be the inconsistent treatment of confounders in the extant studies. For example, adjustment for socioeconomic status (Hackman et al., 2015) and parental depression (Lund et al., 2020) typically attenuates any SLE effects on child cognition in the general population. Another reason may be a lack of consideration for timing and type of exposure (Gabard‐Durnam & McLaughlin, 2019). For example, exposure to mild stressors at critical periods can produce inoculation effects and be cognitively enhancing rather than damaging (e.g., Francesconi et al., 2023). Importantly, the timing of exposure, even to the same stressors, may be crucial for determining future outcomes, as children might adapt and cope differently depending on their neurodevelopmental stage (e.g., preschool vs. school age) (Grant et al., 2006).

The evidence from rodent models is more consistent, clearly indicating that SLE impairs cognitive performance, resulting in memory (Featherstone et al., 2022; Xu et al., 2022) and attention (Kim et al., 2020) problems via several biological mechanisms. These include epigenetic alterations, modification of the autonomic regulation of stress (Oosterman et al., 2010), and activation of the immune system (Schwaiger et al., 2016). For instance, in response to stressor exposure, the human immune system initiates inflammatory responses, in turn producing cognitive symptoms of depression (Colasanto et al., 2020; Pitharouli et al., 2021) since cytokines act as signalling molecules in the central nervous system (Brown et al., 2014). The animal model studies also highlight the key role of timing, severity and chronicity of SLE in determining biological pathways. For example, long‐term exposure to stressful experiences can lead to chronic stimulation of the sympathetic nervous system and to the progressive suppression of some main anti‐inflammatory pathways, such as the Hypothalamic–Pituitary–Adrenal (HPA) axis and the parasympathetic nervous system (Shonkoff et al., 2009) (McEwen & Gianaros, 2010).

In humans too, both long‐term and early‐life stressor exposure have been linked to inflammation, i.e., a pro‐inflammatory signature characterized by elevations, for example, in peripheral C‐reactive protein (CRP), interleukin (IL‐6) and tumour necrosis factor‐*α* (Andersen, 2022; Baumeister et al., 2016). Importantly, exposure to stressors, even if mild, can result in the production of a pro‐inflammatory response. This is especially the case for exposures during a sensitive period, such as early life (Francesconi et al., 2023). In turn, inflammation has been suggested to impact cognition through effects on neurotransmission, synaptic plasticity and branching of dendrites (Andersen, 2022). Via this effect on cognition, inflammation has also been linked to psychiatric disorders such as depression (Osimo et al., 2020), since cognitive deficits are characteristic of psychiatric disorders. For example, working memory, executive function, and episodic memory are impaired in both depression and bipolar disorder (Millan et al., 2012). Given the evidence from both population and animal studies, it is therefore plausible that there is a causal relationship between SLE and cognition in humans, which is mediated by inflammation.

We carried out this study to test this link in childhood in the general population. Our hypothesis was that lifetime stressor exposure would be related to poor cognitive functioning in children (aged 10–11 years old) via inflammation (IL‐6 and CRP), measured at age 9 years. To test for outcome specificity in this association, we measured cognitive functioning broadly, with instruments assessing children's working memory, selective attention, attentional control, response inhibition, communication problems and social cognition. Our study therefore tested the link between SLE and child cognitive functioning in the general population as well as its mediation via inflammation. By focusing on the general child population, it also filled other gaps. There is currently little evidence of a link between inflammation and cognitive functioning in the general child population (e.g., Kokosi et al., 2021) despite much evidence on the long‐term effects of inflammation on the brain during the foetal and the neonatal period when inflammation plays a major role in determining the risk of a variety of neurological disorders (O'Shea et al., 2013). In children, studies examining the link tend to be on special populations, such as those with obstructive sleep apnea and sleep‐disordered breathing (Gozal et al., 2008; Tauman et al., 2004).

## METHODS

### Participants

**FIGURE 1:**
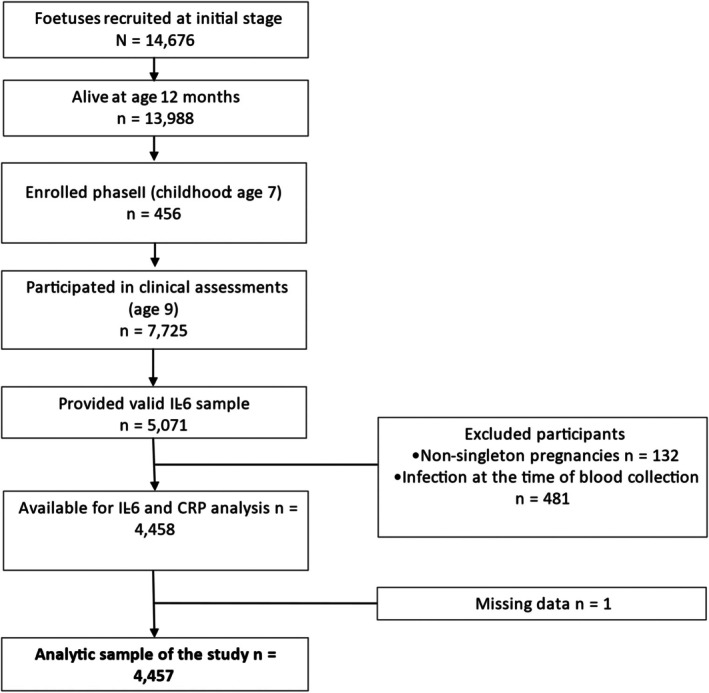
Flowchart of the selection process of the study participants.

The sample population for this study was derived from the Avon Longitudinal Study of Parents and Children (ALSPAC; http://www.bristol.ac.uk/alspac/researchers/our‐data/). ALSPAC is an ongoing prospective population‐based study (Golding, 2004). The initial stage of recruitment invited pregnant women with expected due dates between 1 April 1991 and 31 December 1992, living in the study area, Avon, to participate. Of those interested in participating, 85% were eligible (Golding, 2004). Initially, 14,541 pregnant women, totalling 14,676 foetuses, were recruited from the 15,717 who were eligible and showed interest (Boyd et al., 2013; Fraser et al., 2013). From these pregnancies, 14,062 children with known outcomes were live‐born and 13,988 were alive at 1 year of age. The second recruitment phase targeted children aged 7 years who would have been previously eligible at birth (Boyd et al., 2013). This allowed another 456 children to enrol in the postnatal phase at age 7 and a further 257 during phase 3 of recruitment at age 8 (Boyd et al., 2013). The total sample size for analyses using any data collected after the age of seven is therefore 15,447 pregnancies, resulting in 15,658 foetuses. Of these, 14,901 children were alive at 1 year of age (Boyd et al., 2013). Ethical approval for ALSPAC was granted by the ALSPAC Ethics and Law Committee and Local Research Ethics Committees. Participants gave informed consent for the collection and use of their data and were not provided with financial compensation for partaking in the study (more information at www.alspac.bris.ac.uk). A total of 7725 participated in the clinic assessments at age 9 (62% of those invited). Our study's analytic sample (*n* = 4457; see flowchart in Figure 1) comprised children born from singleton pregnancies who had valid data on inflammatory markers from the clinic assessment at age 9 and who did not report an infection at the time of the blood sample collection.

### Measures

#### Inflammation, age 9 years

This was measured from blood samples of non‐fasting participants during a clinic visit at age 9. Inflammatory markers were assayed following a median storage of 7.5 years, during which there were no freeze–thaw cycles. Although the samples were frozen for an extensive period, which could influence the quality of enzyme‐linked immunosorbent assay (ELISA), previous ALSPAC studies have shown that immune markers can be reliably measured. IL‐6 (pg/mL) was measured by ELISA (R&D Systems: Minneapolis, MN, United States) and high‐sensitivity CRP (mg/L) was measured by automated particle‐enhanced immunoturbidimetric assay (Roche: Welwyn Garden City, Hertfordshire, United Kingdom). All inter‐assay coefficients of variation were low, less than 5%.

#### Stressful life events (SLE), ages 6 months to 8.5 years

Data on upsetting childhood life events were collected from the child's mother. Information was recorded at seven timepoints, i.e., at 18 months (recording events since the child was 6 months old), 30 months (events since 18 months old), 42 months (events since 30 months old), 57 months (events since 3 years old), 69 months (events during the past 15 months), 81 months (events since 5 years old) and 103 months (events since 7 years old). The list of events is a standard checklist of life events (both common and rare) compiled for ALSPAC using previous checklists (Barnett et al., 1983; Brown et al., 1973; Brown, Sklair, Harris, & Birley, 2009; Honnor et al., 1994). The questions asked at all seven timepoints are presented in Table 1. As can be seen, an exception to the questions presented to the mothers was at 81 months. From this age onwards, it was no longer applicable to ask about the child starting nursery. Additional questions were also asked at the later timepoints, marking the child's transition to school. In total, 15 events were measured at ages 18, 30, and 42 months; 16 events at 57 and 69 months; 18 events at 81 months; and 17 events at 103 months (at this last timepoint the question about starting school was dropped as non‐applicable). For our analysis, we pooled the seven summative scores of events. Therefore, lifetime stressor exposure was the sum of SLE across childhood (see Table 2).

**TABLE 1:** List of stressful life events presented to the mothers.

Stressful life events
*Items asked at all timepoints*
Child was taken into care
A pet died
Child moved home
Child experienced a shock or a fright
Child was physically hurt by someone
Child was sexually abused
Child was separated from their mother for at least a week
Child was separated from their father for at least a week
Child acquired a new parent
Child had a new brother or sister
Child was admitted to hospital
Child had changed carer or care giver
Child was separated from someone else
Child started creche or nursery
Child experienced another upsetting event
Additional items asked at 57, 69 and 81 months
Child started school
Additional items asked at 81 months
Somebody in the family died
Child started a new school/kindergarten
Child lost their best friend
Additional items asked at 103 months
Child started a new school

*Note*: Mothers responded to the questions on behalf of their child. Most questions were asked at all seven of the time points used in this study. Additional questions asked only at later time points are presented separately at the bottom of the table.

**TABLE 2:** Descriptive statistics of the main variables from the sample (*N* = 4457).

Continuous variables	*N*	Mean (SD)
Stressful life events (SLE) in childhood	4457	8.98 (5.28)
SLE 18 months	3998	0.95 (1.03)
SLE 30 months	3838	1.39 (1.28)
SLE 42 months	3837	1.64 (1.25)
SLE 57 months	3761	2.36 (1.38)
SLE 69 months	3619	1.44 (1.25)
SLE 81 months	3615	1.35 (1.38)
SLE 103 months	3727	1.52 (1.37)
Maternal stressful events	3890	5.50 (2.91)
Interleukin 6 (pg/mg)	4457	1.21 (1.47)
C‐reactive protein (mg/L)	4457	0.63 (1.95)
Working memory	3759	3.43 (0.85)
Response inhibition (150 ms delay)	3753	12.10 (3.07)
Response inhibition (250 ms delay)	3753	13.66 (2.67)
Verbal ability	3799	151.12 (7.42)
Social cognition	3717	2.27 (3.44)
Selective attention	3780	3.55 (1.25)
Attentional control	3659	18.46 (1.31)
BMI	4406	17.59 (2.77)
Maternal depression	4033	6.71 (4.85)

Categorical variables	*N*	%
Sex (female)	4457	49.23
Ethnicity (white)	4045	95.90
Maternal education	4099	
CSE	536	13.08
Vocational	321	7.83
O level	1446	35.28
A level	1100	26.84
Degree	696	16.98

*Note*: The values presented for IL‐6 and CRP are raw.

#### Working memory, age 10 years

This was measured with the Counting Span Task, a reliable and valid tool to assess working memory (Conway et al., 2005). The task involves red and blue dots presented on a computer screen. The child is asked to point to and count the number of red dots out loud (the processing component). Children are shown: (a) two practice sets of two screens, (b) three sets of two screens, (c) three sets of three screens, (d) three sets of four screens, and finally, (e) three sets of five screens. After each set, the child is asked to recall the number of red dots seen on each screen in the order they were presented within that set (the storage component). In ALSPAC, all children worked through all the sets regardless of their overall performance. A child's working memory span was calculated automatically by the computer programme, on the basis of the number of correctly recalled sets, weighted by the number of screens within each set. The maximum score a child could achieve was 5 (i.e., all correct). In this study, we used the span score, which is the main outcome measure for this task.

#### Response inhibition, age 10 years

This was measured with a stop signal task. The assessment involved a computer and two stimulus boxes, labelled X and O. First, the primary task trial, the practice round, was conducted. The child watched a smiley face on the screen. When either an O or an X appeared, they had to press the corresponding box. The computer used the time taken in the primary task trial to calculate the child's reaction time. The first 30 trials allowed the child to become familiar with the test before conducting the stop signal task. This involved the repetition of the task, except with bleeps sounding on random trials. On the occasions when the bleep sounded, the child had to resist pressing the stimulus box. Therefore, the child was required to inhibit the already initiated movement. The second block of trials consisted of 24 practice trials, comprising 8 primary task trials and 16 stop signal trials. Two further blocks of trials, the experimental blocks, were completed. These consisted of 48 trials in total, of which 32 were without and 16 were with bleeps. Bleeps sounded either 150 or 250 ms before the child's reaction time, calculated from the primary task. Here the measures used to assess performance were the number of stop signal trials correct at 250 ms delay and at 150 ms delay. Therefore, higher scores indicate better response inhibition.

#### Verbal ability, age 9 years

This was measured with the mother‐reported Children's Communication Checklist (CCC), a measure of pragmatic impairments in language and communication (Bishop, 1998). The CCC version adapted in ALSPAC included 53 of the 70 items (scored on a 3‐point scale) and 7 of the 9 subscales, with higher scores suggesting better language and communication. Here, the prorated pragmatic aspects of communication score, provided by ALSPAC, were used.

#### Social cognition, age 10 years

This was measured with the 12‐item mother‐reported Social and Communication Disorder Checklist (SCDC) (Skuse et al., 1997). Items include ‘Not aware of other peoples’ feelings', ‘Does not seem to understand social skills’, and ‘Cannot follow a command unless it is carefully worded’. Each item is scored 0–2, with higher scores suggesting poorer social cognition. The SCDC is considered reliable and valid, and a useful tool both for examining autistic traits in the population and for screening for individual cases (Skuse et al., 2005).

#### Selective attention, age 11 years

This was measured using an adapted version of the Tests of Everyday Attention for Children (TEACh) (Manly et al., 2001). The selective attention task examines the ability to separate important information from distractions. The task required children to identify pairs of identical spaceships from amongst a collection of many spaceship images. First, a practice trial was completed before proceeding to the test. By matching 20 pairs of spaceships on a larger sheet, the child's accuracy and time taken to complete the task were recorded. This produced the unadjusted score. Next, the motor task was conducted, which involved repeating the previous task; however, only the identical pair of spaceships remained, allowing the speed of the motor movements to be measured and controlled for. The final score, used here, was calculated from the time taken in the first test to match up the spaceships, with the motor score from the second test subtracted to adjust for the motor movements. Therefore, fast and accurate responses produce lower scores, indicating better attentive abilities.

#### Attentional control, age 11 years

This was measured with the ‘opposite world’ task, another subtask from TEACh. Similar to the Stroop test (Stroop, 1935), the opposite worlds task requires the participants to process two stimuli simultaneously, requiring one to inhibit the other. In the control task, ‘same world’, the child is required to read a series of 24 numbers, either 1 or 2, in the shortest amount of time they can. The tester points at the numbers as the child progresses. Next, in the ‘opposite world’ section, the child repeats the test, this time needing to say 2 when they see 1 and vice versa. During the ‘opposite world’ task, the child must inhibit the response already initiated to give the correct answer. When the assessment was conducted in ALSPAC, the tester demonstrated how the rules worked, and then the child was given a practice before the actual trials. There were two rounds of both ‘opposite world’ and ‘same world’ trials. Mean times for both ‘same world’ and ‘opposite world’ trials were calculated. Here we used the normative score, provided by ALSPAC, for the ‘opposite world’ trials.

#### Confounders

We adjusted for the following confounders (variables associated with both exposures and outcomes): maternal stressful life events during pregnancy, ethnicity, sex, socioeconomic status, and maternal depression. We also adjusted for body mass index (BMI) at the time inflammation was measured, as is standard. Stressful life events during pregnancy were measured with a checklist of 42 events, administered twice, at 18 weeks gestation and 8 weeks post‐partum. The full list of events can be found in Table S3. Ethnicity was dichotomously recorded following birth as “white” or “non‐white”, as was sex, which was recorded as female or male. Socioeconomic status was measured with mother's highest qualification at 32 weeks of gestation. Maternal depression, also at 32 weeks of gestation, was measured using the Edinburgh Post‐Natal Depression Scale (Cox et al., 1987). This screening tool comprises 10 questions, each answered on a scale of 0–3, about the mother's feelings during the past week. Finally, BMI (weight (kg)/height (m^2^)) was measured during the clinic visit at age 9 years, when data on inflammation were also collected.

### Analytic strategy

First, descriptive statistics for all the study variables were examined, followed by the correlations between the main study variables (exposure, mediators and outcomes). To test for mediation by inflammation, seemingly unrelated regression models were fitted before and after adjustment for confounders. Seemingly unrelated regressions (SUR) refer to a statistical method used to estimate multiple regression equations where the error terms of the equations may be correlated, but the dependent variables are not. This approach allows for more efficient estimation when dealing with multiple outcomes simultaneously, as used in this mediation analysis for IL‐6 and CRP in relation to cognitive outcomes. In the SUR models we fitted, IL‐6 and CRP were log‐transformed to reduce skewness. Multivariate imputation by chained equations (MICE) was used to accommodate data missingness (Azur et al., 2011). Data for stressful life events across childhood, sex, IL‐6, and CRP were complete and not imputed. All analysis was performed in STATA (18.0).

## RESULTS

### Descriptive statistics

The descriptive statistics are shown below in Table 2. As can be seen, the mean number of SLE across childhood was 9 with a standard deviation of 5, indicating great variability in exposure. The sample was ethnically homogenous (96% white), but almost equally divided by sex (49% female). Overall, low levels of inflammatory markers were observed, as expected. Moreover, Table S2 presents the frequency of each event measured across childhood. As expected, the most severe events, such as sexual abuse, were less frequent, while milder events occurred more often. For instance, over half of the sample reported experiencing the death of a pet.

### Bivariate correlations

Table S1 displays the bivariate correlations between the main study variables (exposure, mediators, and outcomes). SLE had a positive correlation with impaired working memory and impaired social cognition, and a negative correlation with verbal ability. The two inflammatory markers, IL‐6 and CRP, were inter‐correlated, as expected. However, neither was significantly correlated with childhood SLE. With respect to associations between mediators and outcomes, CRP had negative correlations with working memory and verbal ability, but there was no other statistically significant association.

### Regression models

Table 3 presents the results from the seemingly unrelated regressions using imputed data. For each cognitive outcome, models are presented both before and after adjusting for confounders. As can be seen, *working memory* had a negative association with both IL‐6 and CRP, even after adjustment, but the indirect effect was nonsignificant. SLE was not related to working memory after adjustment. By contrast, there was an inverse association between SLE and *verbal ability* even after adjustment for confounders. Indirect effects, via either IL‐6 or CRP, however were nonsignificant. SLE was also positively related to *social cognition impairment* even after adjustment, but, again, indirect effects, via IL‐6 or CRP, were not significant. SLE and CRP were not related to *selective attention impairment*, unlike IL‐6, which was positively related. Finally, on both *response inhibition* and *attentional control* there were nonsignificant effects of both inflammation and SLE.

**TABLE 3:** Mediation models^a^ showing regression coefficients, *p*‐values, and confidence intervals at 95% for the paths investigated as well as the indirect and total effect (3sf).

	Coefficient	*p* > |z|	95% CI
SLE → IL‐6 → working memory (WM)			
Unadjusted model			
SLE → IL‐6	0.00	.51	−0.00, 0.01
SLE → WM	0.01	.01	0.00, 0.01
IL‐6 → WM	−0.05	.002	−0.09, −0.02
Indirect effect	−0.00	.53	−0.00, 0.00
Total effect	0.01	.01	0.00, 0.01
Adjusted model			
SLE → IL‐6	0.00	.52	−0.00, 0.01
SLE → WM	0.00	.44	−0.00, 0.01
IL‐6 → WM	−0.04	.01	−0.08, −0.01
Indirect effect	−0.00	.54	−0.00, 0.00
Total effect	0.00	.46	−0.00, 0.01
SLE → CRP → working memory (WM)			
Unadjusted model			
SLE → CRP	0.00	.78	−0.01, 0.01
SLE → WM	0.01	.01	0.00, 0.01
CRP → WM	−0.04	.001	−0.07, −0.02
Indirect effect	−0.00	.78	−0.00, 0.00
Total effect	7.34 × 10^−3^	.005	0.00, 0.01
Adjusted model			
SLE → CRP	0.00	.46	−0.00, 0.01
SLE → WM	0.00	.44	−0.00, 0.01
CRP → WM	−0.04	.003	−0.07, −0.01
Indirect effect	−0.00	.48	−0.00 0.00
Total effect	0.00	.46	−0.00, 0.01
SLE → IL‐6 → verbal ability (VA)			
Unadjusted model			
SLE → IL‐6	0.00	.51	−0.00, 0.01
SLE → VA	−0.05	.05	−0.09, 0.00
IL‐6 → VA	−0.31	.03	−0.59, −0.03
Indirect effect	−0.00	.53	−0.00, 0.00
Total effect	−0.045	.05	−0.09, −0.00
Adjusted model			
SLE → IL‐6	0.00	.52	−0.00, 0.01
SLE → VA	−0.05	.04	−0.1, −0.00
IL‐6 → VA	−0.17	.23	−0.44, 0.11
Indirect effect	−0.00	.58	−0.00, 0.00
Total effect	−0.05	.04	−0.1, −0.00
SLE → CRP → verbal ability (VA)			
Unadjusted model			
SLE → CRP	0.00	.78	−0.01, 0.01
SLE → VA	−0.05	.05	−0.09, −0.00
CRP → VA	−0.18	.09	−0.38, 0.03
Indirect effect	−0.00	.79	−0.00, 0.00
Total effect	−0.05	.05	−0.09, −0.00
Adjusted model			
SLE → CRP	0.00	.46	−0.00, 0.01
SLE → VA	−0.05	.04	−0.1, −0.00
CRP → VA	−0.12	.3	−0.34, 0.11
Indirect effect	−0.00	.56	−0.00, 0.00
Total effect	−0.05	.04	−0.1, −0.00
SLE → IL‐6 → social cognition impairment (SCI)			
Unadjusted model			
SLE → IL‐6	0.00	.51	−0.00, 0.01
SLE → SCI	0.05	<.001	0.03, 0.07
IL‐6 → SCI	0.02	.73	−0.11, 0.15
Indirect effect	0.00	.79	−0.00, 0.00
Total effect	0.05	<.001	0.03, 0.07
Adjusted model			
SLE → IL‐6	0.00	.52	−0.00, 0.00
SLE → SCI	0.04	<.001	0.02, 0.06
IL‐6 → SCI	−0.00	.95	−0.13, 0.13
Indirect effect	0.00	.95	−0.00, 0.00
Total effect	0.04	<.001	0.02, 0.06
SLE → CRP → social cognition impairment (SCI)			
Unadjusted model			
SLE → CRP	0.00	.78	−0.01, 0.01
SLE → SCI	0.05	<.001	0.03, 0.07
CRP → SCI	0.04	.43	−0.06, 0.14
Indirect effect	0.00	.81	−0.00, 0.00
Total effect	0.05	<.001	0.03, 0.07
Adjusted model			
SLE → CRP	0.00	.46	−0.00, 0.01
SLE → SCI	0.04	<.001	0.02, 0.06
CRP → SCI	0.03	.59	−0.08, 0.14
Indirect effect	0.00	.69	−0.00, 0.00
Total effect	0.04	<.001	0.02, 0.06
SLE → IL‐6 → selective attention impairment (SAI)			
Unadjusted model			
SLE → IL‐6	0.00	.5	−0.00, 0.001
SLE → SAI	−0.01	.19	−0.01, 0.00
IL‐6 → SAI	0.02	.38	−0.02, 0.07
Indirect effect	0.00	.62	−0.00, 0.00
Total effect	−0.01	.2	−0.01, 0.00
Adjusted model			
SLE → IL‐6	0.00	.52	−0.00, 0.01
SLE → SAI	−0.00	.43	−0.01, 0.00
IL‐6 → SAI	0.05	.04	0.00, 0.1
Indirect effect	0.00	.55	−0.00, 0.00
Total effect	−0.00	.43	−0.01, 0.00
SLE → CRP → selective attention impairment (SAI)			
Unadjusted model			
SLE → CRP	0.00	.78	−0.01, 0.01
SLE → SAI	−0.01	.2	−0.01, 0.00
CRP → SAI	0.00	1	−0.04, 0.04
Indirect effect	0.00	1	−0.00, 0.00
Total effect	−0.01	.2	−0.01, 0.00
Adjusted model			
SLE → CRP	0.00	.46	−0.00, 0.01
SLE → SAI	−0.003	.42	−0.01, 0.00
CRP → SAI	0.03	.14	−0.01, 0.07
Indirect effect	0.00	.52	−0.00, 0.00
Total effect	−0.00	.43	−0.01, 0.00
SLE → IL‐6 → response inhibition (RI)*			
Unadjusted model			
SLE → IL‐6	0.00	.51	−0.00, 0.01
SLE → RI	−0.00	.96	−0.02, 0.02
IL‐6 → RI	−0.09	.14	−0.20, 0.03
Indirect effect	−0.00	.56	−0.00, 0.00
Total effect	−0.00	.95	−0.02, 0.02
Adjusted model			
SLE → IL‐6	0.00	.25	−0.00, 0.01
SLE → RI	0.00	.98	−0.02, 0.02
IL‐6 → RI	−0.11	.06	−0.23, 0.01
Indirect effect	−0.00	.34	−0.00, 0.00
Total effect	−0.00	.99	−0.02, 0.02
SLE → CRP → response inhibition (RI)*			
Unadjusted model			
SLE → CRP	0.00	.78	−0.01, 0.01
SLE → RI	−0.00	.95	−0.02, 0.02
CRP → RI	−0.04	.32	−0.13, 0.04
Indirect effect	−0.00	.8	−0.00, 0.00
Total effect	−0.00	.95	−0.02, 0.02
Adjusted model			
SLE → CRP	0.00	.46	−0.00, 0.01
SLE → RI	0.00	.87	−0.02, 0.02
CRP → RI	−0.07	.14	−0.170 0.02
Indirect effect	−0.00	.52	−0.00, 0.00
Total effect	0.00	.88	−0.02, 0.02
SLE → IL‐6 → attentional control (AC)			
Unadjusted model			
SLE → IL‐6	0.00	.51	−0.00, 0.00
SLE → AC	−0.00	.96	−0.01, 0.01
IL‐6 → AC	−0.01	.78	−0.06, 0.04
Indirect effect	−0.00	.82	−0.00, 0.00
Total effect	−0.00	.95	−0.01, 0.01
Adjusted model			
SLE → IL‐6	0.00	.52	−0.00, 0.01
SLE → AC	−0.00	.31	−0.01, 0.00
IL‐6 → AC	−0.00	.85	−0.06, 0.05
Indirect effect	−0.00	.87	−0.00, 0.00
Total effect	−0.00	.31	−0.01, 0.00
SLE → CRP → attentional control (AC)			
Unadjusted model			
SLE → CRP	0.00	.78	−0.00, 0.01
SLE → AC	−0.00	.96	−0.01, 0.01
CRP → AC	−0.02	.32	−0.06, 0.02
Indirect effect	−0.00	.8	−0.00, 0.00
Total effect	−0.00	.95	−0.01, 0.01
Adjusted model			
SLE → CRP	0.00	.46	−0.00, 0.01
SLE → AC	−0.00	.31	−0.01, 0.00
CRP → AC	−0.02	.25	−0.07, 0.02
Indirect effect	−0.00	.55	−0.00, 0.00
Total effect	−0.00	.31	−0.01, 0.00

*Note*: *For reasons of economy, only results for a 150 ms delay are presented as results for a 250 ms delay (available on request) were very similar.

^a^ Seemingly unrelated regressions (SUR) are used to estimate multiple regression equations where the error terms may be correlated, allowing for more efficient estimation of the mediation models for IL‐6 and CRP in relation to cognitive outcomes.

## DISCUSSION

This general‐population study investigated whether the accumulation of stressor exposure since infancy is related to poor cognitive functioning in late childhood via inflammation (IL‐6 and CRP). Its strengths include (a) the prospective measurement of a wide range of stressful experiences—from the rare and extreme (e.g., sexual abuse) to the normative and mundane (e.g., started nursery); (b) the inclusion of a range of cognitive measures capturing difficulties in both communication and executive function, and (c) a stringent adjustment for confounders. While it did not find evidence for inflammation mediating the effect of stressful life events on cognitive functioning, some of the individual associations observed are important and warrant attention.

One of the principal findings was that inflammation was associated with executive function impairments, even after confounder adjustment. For example, both IL‐6 and CRP at age 9 were negatively associated with working memory at age 10, even after controlling for children's exposure to stressful events since infancy but also mothers' exposure to stressors throughout pregnancy, maternal depression, socioeconomic status, ethnicity, sex and BMI. This finding is in line with existing research showing that the upregulation of several immune markers is negatively associated with working memory (Lee et al., 2016), likely due to inflammation disrupting mechanisms responsible for memory consolidation and synaptic plasticity. In fact, a recent study showed that mother's IL‐6 during pregnancy was related to her child's working memory (Rudolph et al., 2018), suggesting that inflammation during the gestational period can interfere with neurodevelopment. IL‐6 (but not CRP) was also related to difficulties in selective attention. Recently, there has been much interest in the involvement of the immune system in the aetiology of ADHD (Misiak et al., 2022; Schnorr et al., 2024), the pathophysiology of which remains largely unknown (Misiak et al., 2022). Nonetheless, we must acknowledge that the size of the effect we found was rather small. Further research is required. Clarifying if there is a particular developmental window when inflammation is particularly damaging may be very useful, scientifically and practically.

On the other hand, difficulties in social cognition (as measured with the SCDC) and social communication (as measured with the CCC) were not related to inflammation, although they were robustly related to stressful life events (SLE). As with the other associations in this study, these were small, suggesting that factors other than stressor exposure in childhood are at play. Nonetheless, they are substantively important. Both the CCC and the SCDC assess behaviours symptomatic of ASD. Identifying the mechanisms underlying ASD is valuable for improving available treatments but also screening methods, which tend to show low sensitivity, particularly in young children (Lord et al., 2018).

In conclusion, while small in size, the associations we established have significant implications for both research and practice. The link between inflammation and impairments in executive function, particularly working memory and selective attention, suggests that even modest inflammatory responses could disrupt cognitive development, in line with some existing research (Lee et al., 2016; Misiak et al., 2022). This underscores the potential value of targeting inflammation as a means of supporting cognitive functioning in childhood, when brain development is very malleable (Rudolph et al., 2018). Additionally, the robust link between stressful life events and social communication difficulties highlights the lasting impact of early adversity on social functioning, consistent with findings that stress exposure can impair social adjustment and contribute to difficulties in communication (Lord et al., 2018). This, in turn, points to the importance of reducing stressor exposure in childhood as a means of enhancing social adjustment and thus promoting mental health and well‐being. Ultimately, our study suggests that a dual focus on reducing inflammation and managing stress could improve children's executive functioning, whereas preventing early‐life stress could improve their social skills.

While these are important findings, they are limited in several ways. First, the data on SLE rely on the assumption that the mother is a reliable reporter of her child's level of stressor exposure. Second, information about the perceived impact of events may have been pertinent, especially given the focus on inflammation. Third, inflammation was measured once. Fourth, ALSPAC, like any prospective cohort study, suffers from significant sample loss, which is also non‐random. This, in turn, introduces a self‐selection bias to the sample population. Finally, given that this study was observational, causality from the relationships established cannot be determined. This is a particularly important limitation. Evidence from experimental studies in older adults (Reichenberg et al., 2001) and rodents (Chen et al., 2008) suggests that acute systemic inflammation directly impairs cognitive functions such as working memory. Conversely, a longitudinal analysis of human participants showed that early cognitive functioning (at age 11 years) determined lifetime pro‐inflammatory exposures, which consequently led to cognitive decline at an older age (Luciano et al., 2009). Our own longitudinal study can only suggest that there is a relationship between inflammation and executive functioning in middle childhood. We are not able to draw any conclusions about causality or whether a reverse association exists.

Taken together, our findings suggest that the direct effects of SLE and inflammation on cognitive and social functioning in the general child population warrant a serious consideration of specificity. In our study, inflammation was related to executive function impairments, and SLE were related to social communication difficulties. Importantly, these associations were robust to adjustment for confounders. Therefore, if causal, they suggest that inflammation, even if not produced by SLE (at least in the way they were measured here), can have very specific impacts. Thus, reducing inflammation could improve executive functioning, the prerequisite to any purposeful and goal‐directed action. They also suggest that SLE, even after confounder adjustment, can impair social communication, the prerequisite to social adjustment. This, in turn, indicates that reducing stressor exposure in childhood can improve social functioning both in the short and in the long term.

In terms of future directions, we think that studies testing SLE effects should consider information about both the timing and the severity of the exposure (Francesconi et al., 2023). One study, for example, found that experiences of most types of stressful events during early childhood, before age 3 years, were associated with DNA methylation (Dunn et al., 2019). By contrast, in middle childhood, only experiences of extreme adversity, such as sexual abuse, produced an epigenetic response. This shows that the extent of epigenetic change following a stressful event depends on the child's stage of development. However, the type of event may be important too. For example, a recent study, also using ALSPAC (Francesconi et al., 2023), found that undergoing *mild* stressful events early in childhood (across ages 1 to 5 years old) was associated with better cognitive performance (as measured by IQ scores in adolescence), suggesting that mild SLE early in childhood may actually be cognitively enhancing.

## CONCLUSIONS

This general‐population study investigated the role of inflammation in mediating the effect of cumulative SLE exposure on executive function and social communication in late childhood. Inflammation did not mediate the link between SLE and either of these outcomes. Nonetheless, inflammation was related to executive function impairments, and cumulative SLE exposure was related to social communication difficulties. Despite the small effects found, these associations warrant further investigation.

## AUTHOR CONTRIBUTIONS

**Izabella Polgar‐Wiseman:** Investigation; writing – original draft; formal analysis. **Marta Francesconi:** Conceptualization; writing – review and editing; supervision; methodology; data curation. **Eirini Flouri:** Conceptualization; funding acquisition; methodology; project administration; supervision; data curation; writing – review and editing.

## CONFLICT OF INTEREST STATEMENT

None. The authors report no biomedical financial interests or other potential conflicts of interest.

## Supporting information


Table S1.
Table S2.
Table S3.

## Data Availability

Data sharing is not applicable to this article as no new data were created or analyzed in this study.
