# Supplementary material for: Cumulative stressor exposure and cognitive functioning in late childhood: The role of inflammation
Source: Br J Dev Psychol. 2025 Mar 23;43(4):843–59. doi: 10.1111/bjdp.12557 (PMC12505829; doi:10.1111/bjdp.12557)
Supplement: Supplementary file 1 — Table S1. Table S2. Table S3. [file BJDP-43-843-s001.docx]

**Table 1**

*Bivariate Correlations of the Main Variables and Confounders (Exposures, Mediators, and Outcomes)*

|  | 1. | 2. | 3. | 4. | 5. | 6. | 7. | 8. | 9. | 10. | 11. | 12. | 13. | 14. | 15. | 16. |
| --- | --- | --- | --- | --- | --- | --- | --- | --- | --- | --- | --- | --- | --- | --- | --- | --- |
| 1. | 1 |  |  |  |  |  |  |  |  |  |  |  |  |  |  |  |
| 2. | 0.30** | 1 |  |  |  |  |  |  |  |  |  |  |  |  |  |  |
| 3. | 0.00 | 0.02 | 1 |  |  |  |  |  |  |  |  |  |  |  |  |  |
| 4. | 0.02 | -0.00 | 0.40** | 1 |  |  |  |  |  |  |  |  |  |  |  |  |
| 5. | 0.05** | 0.04* | -0.03 | -0.04** | 1 |  |  |  |  |  |  |  |  |  |  |  |
| 6. | -0.00 | -0.03 | -0.02 | 0.01 | 0.12** | 1 |  |  |  |  |  |  |  |  |  |  |
| 7. | -0.00 | -0.01 | -0.07 | 0.00 | 0.08** | 0.72** | 1 |  |  |  |  |  |  |  |  |  |
| 8. | -0.04** | -0.12** | -0.02 | -0.05** | 0.14** | 0.07** | 0.06** | 1 |  |  |  |  |  |  |  |  |
| 9. | 0.01** | 0.09** | 0.01 | 0.02 | -0.04* | -0.06** | -0.05** | -0.46** | 1 |  |  |  |  |  |  |  |
| 10. | -0.02 | -0.028 | 0.01 | 0.01 | -0.15** | -0.13** | -0.08** | -0.15** | 0.11** | 1 |  |  |  |  |  |  |
| 11. | -0.01 | 0.00 | 0.00 | 0.00 | 0.18** | 0.13** | 0.11** | 0.15** | -0.10** | -0.33** | 1 |  |  |  |  |  |
| 12. | 0.02 | 0.05** | 0.02 | -0.01 | 0.00 | 0.00 | 0.028 | -0.02 | 0.02 | -0.01 | -0.00 | 1 |  |  |  |  |
| 13. | -0.01 | 0.02 | 0.07** | 0.05** | 0.02 | 0.03 | 0.05** | 0.08** | -0.07** | -0.20** | 0.11** | -0.00 | 1 |  |  |  |
| 14. | 0.00 | 0.02 | 0.12** | 0.21** | -0.0328* | -0.01 | -0.00 | -0.05** | 0.04* | 0.08 | -0.03* | 0.04* | 0.08** | 1 |  |  |
| 15. | 0.17** | 0.07** | -0.03 | -0.06** | 0.17** | 0.02 | 0.01 | 0.21** | -0.04** | -0.05** | 0.09** | 0.00 | 0.00 | -0.07** | 1 |  |
| 16. | 0.07** | 0.26** | -0.01 | 0.02 | -0.05** | -0.05** | -0.03 | -0.22** | 0.16** | 0.02 | -0.04* | 0.05** | 0.03 | 0.03* | -0.13** | 1 |

*Notes.* 1. Stressful Life Events; 2. Maternal Stressful Life Events during Pregnancy; 3. IL-6 (raw data); 4. CRP (raw data); 5. Working Memory; 6. Response Inhibition (250 ms); 7. Response Inhibition (150 ms); 8. Verbal Ability; 9. Social Cognition; 10. Selective Attention; 11. Attentional Control; 12. Ethnicity; 13. Sex; 14. BMI; 15. Maternal Education; 16. Maternal Depression (*p < .05; **p < .01)

| **Table 2.** *Occurrence of stressful (upsetting) life events across childhood* | |
| --- | --- |
|  | |
| **Events** | **N (%)** |
| Child was taken into care | 13 (0.30) |
| A pet died | 2,659 (61.84) |
| Child moved home | 2,282 (53.21) |
| Child experienced a shock or a fright | 1,486 (34.58) |
| Child was physically hurt by someone | 563 (13.08) |
| Child was sexually abused | 19 (0.44) |
| Child was separated from their mother for at least a week | 1,006 (23.38) |
| Child was separated from their father for at least a week | 1,690 (39.35) |
| Child acquired a new parent | 169 (4.08) |
| Child had a new brother or sister | 2,112 (49.10) |
| Child was admitted to hospital | 1,313 (30.56) |
| Child changed carer or care giver | 843 (19.59) |
| Child was separated from someone else | 656 (15.23) |
| Child started creche or nursery or school | 3,988 (92.85) |
| **Additional items asked at 81 and 103 months** |  |
| Somebody in the family died | 1,296 (32.6) |
| Child lost their best friend | 524 (12.99) |
| *Notes:* This table presents the number (%) of participants who had experienced each event at any time during childhood, i.e., between the ages of 6 and 103 months. | |

| **Table 3.**  *List of Prenatal Stressful Events* | |
| --- | --- |
|  | |
| Your husband/partner died | One of your children died |
| A friend or relative died | One of your children was ill |
| Your husband or partner was ill | A friend or relative was ill |
| You were admitted to hospital | You were in trouble with the law |
| You were divorced | You found that your husband/partner didn’t want your child |
| You were very ill | Your husband/partner lost his job |
| Your husband/partner had problems at work | You had problems at work |
| You lost your job | Your husband/partner went away |
| Your husband/partner was in trouble with the law | You and your husband/ partner separated |
| Your income was reduced | You argued with your husband/partner |
| You argued with your family and friends | You moved house |
| Your partner hurt you physically | You became homeless |
| You had a major financial problem | You got married |
| Your husband/partner was physically cruel to your children | You attempted suicide |
| You were convicted of an offence | You were bleeding and thought you might miscarry |
| You started a new job | You had a test to see if your baby was abnormal |
| You had a result on a test that suggested your baby might not be normal | You were told that you were going to have twins |
| You heard that something that had happened might be harmful to the baby | You tried to have an abortion |
| You took an examination | Your husband/partner was emotionally cruel to you |
| Your husband/partner was emotionally cruel to your children | Your house or car was burgled |
| You had an accident | Other |
| *Notes:* This table presents the list of events measured at 18 weeks’ gestation and 8 weeks post-partum. | |
